# Serum-free alginate-C2C12 cells microcapsule as a model of alternative animal protein source

**DOI:** 10.3389/fnut.2023.1184178

**Published:** 2023-05-11

**Authors:** Jana Scheffold, Per Bruheim, Joachim Sebastian Kjesbu, Mi Jang

**Affiliations:** Department of Biotechnology and Food Science, Norwegian University of Science and Technology, Trondheim, Norway

**Keywords:** serum-free culture, alginate encapsulation, C2C12 cells, alternative animal protein source, metabolomics

## Abstract

Due to the climate change crisis, and environmental impacts of the traditional meat sector, the production of artificial animal protein based on *in vitro* cell culture technology is proposed as an alternative. Furthermore, since traditional animal serum-supplemented cultures pose scientific challenges such as batch variation and contamination risks, artificial animal protein cultures are currently in urgent need of not only serum-free cultures, but also microcarrier culture systems for scalability. However, serum-free microcarrier-based culture system for the differentiation of muscle cells is not available to date. Therefore, we established an edible alginate microcapsules culture system for the differentiation of C2C12 cells in serum-free conditions. Furthermore, metabolites related to central carbon metabolism were profiled based on targeted metabolomics using mass spectrometry. The C2C12 cells cultured in alginate microcapsules displayed high viability throughout 7 days and successfully differentiated within 4 days in serum and serum-free cultures except for AIM-V cultures, which was confirmed by CK activity and MHC immunostaining. Lastly, to the best of our knowledge, this is the first report to compare metabolite profiles between monolayer and alginate microcapsule culture systems. Alginate microcapsule culture showed higher levels of intracellular glycolysis and TCA cycle intermediates, lactate, and the contribution of essential amino acids compared to the monolayer culture. We believe our serum-free alginate microcapsule culture system is adaptable to different species of muscle cells and contributes to future food technology as a proof of concept for the scalability of alternative animal protein source production.

## 1. Introduction

Cultured meat is an emerging field as an alternative source of animal protein that can replace the traditional meat sector, which generates considerable greenhouse gas emissions. Cultured meat is also called artificial meat since it is manufactured in the laboratory based on *in vitro* cell culture techniques (1). The production of cultured meat requires a more efficient large-scale cultivation system (2). Recently, microcarrier-based culture systems have been proposed as a relatively easy solution for upscaling cultured meat production compared to the standard two-dimensional (2D) cell culture, where the surface area is a limiting factor (2). Among various microcarrier-based culture techniques, encapsulation using edible biomaterials is strongly suggested to be more advantageous as it does not require cell dissociation, which is necessary when using non-edible microcarriers (2–4). In addition, given that cultured meat could be the food platform of the future, the choice of microcarrier material source is critical, and selecting edible sources might be a priority. While non-edible microcarrier-based cultures have been reported for the proliferation of primary bovine myoblast cells (5), only a few studies have recently reported on microcarrier-based cell cultures platforms that produce cultured meat using edible material, such as collagen (6) and alginate (7). Unlike collagen derived from animals, alginate is a plant-based material from algae and is widely used as a low-cost ingredient in various food applications (8).

Current *in vitro* culture systems for cultured meat production still rely on animal-derived serum supplementation despite the scientific, technical, and ethical drawbacks (9). In particular, risks of contamination and batch-to-batch variations from animal serum supplementation make it difficult to maintain consistent quality in artificial meat products. Despite the advantages of being environmentally sustainable and cost-saving, serum-free cultures have merely been utilized in the biopharmaceutical industry for the production of vaccines and therapeutic proteins (10), while the field of artificial meat has only recently received significant attention and research has begun to establish serum-free culture (11). Our previous study demonstrated that serum-free media influences cell behavior with respect to cell growth and metabolic characteristics (12). However, it is not clear how serum-free media affects cell behavior in a microcarrier culture system. Moreover, the use of serum-free microcarrier-based culture systems in the field of cultured meat has not yet been reported.

Metabolomics is a comprehensive analytical discipline that profiles metabolites and has been used to investigate food ingredients to assess quality. In particular, it is regarded as a gold standard for the characterization of meat composition and biomarkers discovery in the field of cultured meat (7). Previously, our team established two-dimensional serum-free cultures for mouse myoblast cell lines using commercial serum-free media and further investigated central carbon metabolism (13). Surprisingly, considerably different muscle phenotypes and metabolic profiles were observed between conventional serum-supplemented and serum-free cultures. In addition, 3D spheroid culture typically induces contact inhibition environment, resulting in dramatic metabolic changes such as citrate metabolism. However, until now, few studies have reported on the metabolic difference between 2D and 3D cultures, although 3D cultures exhibit more similar phenotypes to *in vivo* tissue (14).

**Figure 1:**
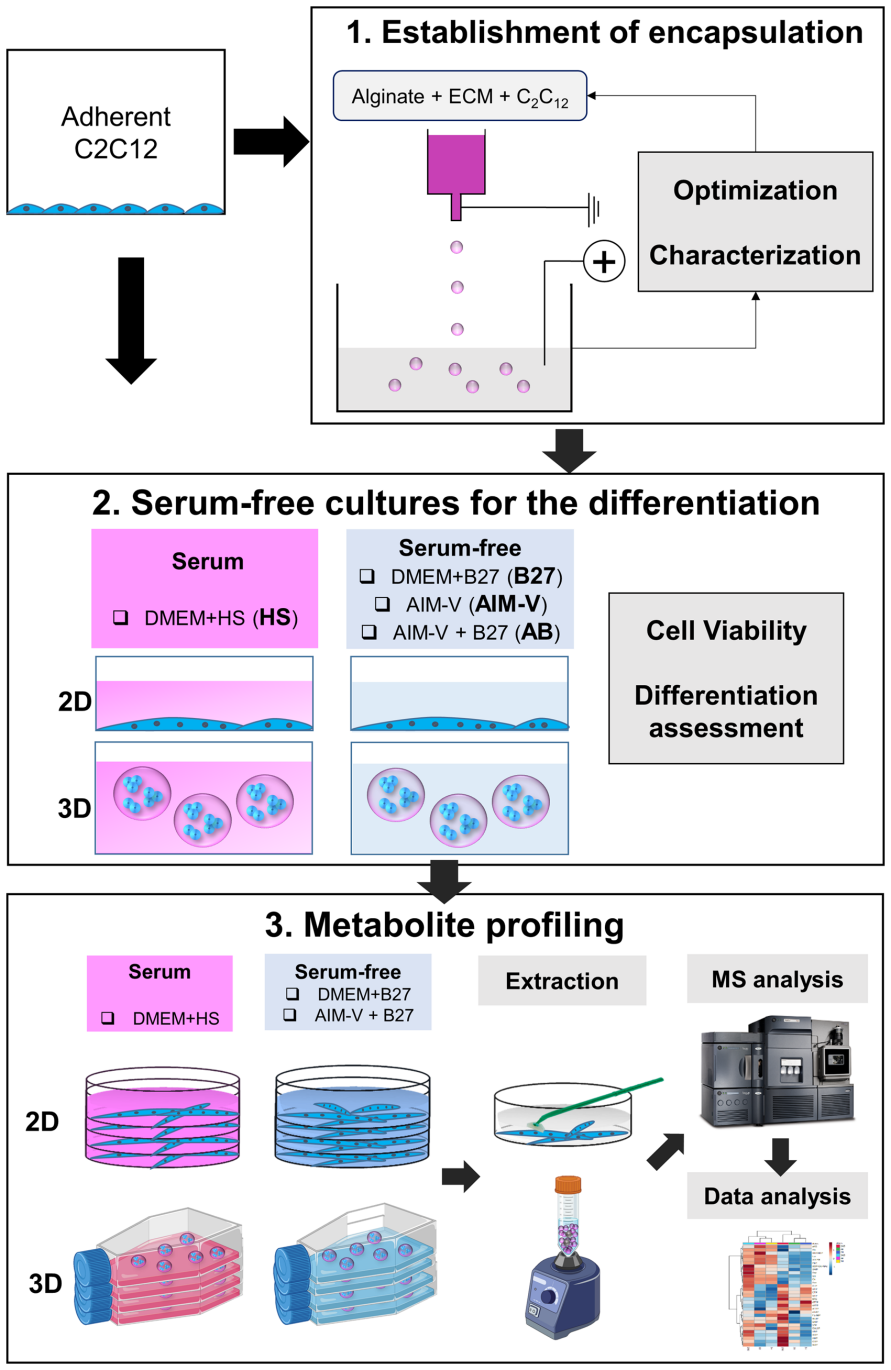
Experimental schematic workflow of the current study for the generation of serum-free alginate microcapsule culture for the differentiation of C2C12 cells and metabolomic study. 1) The establishment of alginate encapsulation procedure. The parameters (voltages and attachment factors) were optimized for a robust alginate beads generation system. Alginate beads encapsulated with cells were characterized regarding morphology, size, and swelling rate. 2) Serum-free cultures were initiated for the differentiation of the C2C12 cells. 4 different media were prepared. i) DMEM supplemented with horse serum (HS), and three types of serum-free media were compared, ii) B27 supplementation in DMEM (B27), iii) AIM-V, and iv) B27 supplementation in AIM-V (AB). Alginate-C2C12 cells microcapsules were cultivated for 7 days and cell viability and differentiation were evaluated. 3) LC/MS-based targeted metabolomics was performed to profile metabolites involved in central carbon metabolism.

In this study, we present a serum-free alginate microcapsule culture using a mouse myoblast cell line (C2C12 cells) as proof of concept for the future animal protein source platform. Exclusively commercially available sources, including media and other materials, were applied to facilitate a user-friendly approach. First, we aimed to establish a robust alginate-encapsulated muscle cell culture system for the differentiation of C2C12 cells. Furthermore, comprehensive metabolic profiling related to central carbon metabolism was conducted to investigate metabolic characteristics influenced by different culture modes and media (Figure 1). We believe that our work is beneficial to tackle the scalability and cost challenges of the large-scale production of cultured meat as an animal protein source and contributes to advancing the competitiveness of cultured meat.

## 2. Methods and materials

### 2.1. Encapsulation of cells in alginate beads

The maintenance of C2C12 cells is described in the Supplementary material. Encapsulation of cells in alginate solution was performed using an electrostatic droplet generator (NTNU in-house fabrication) (15). The mixture solution of cells and alginate was placed in a sterile 10 mL syringe and extruded via a 0.35 mm metal needle with a flow rate of 10 mL/h by applying 5.5 kV (Final fixed voltage after optimization). The collected C2C12 cells were mixed with 1.05% of medium viscosity alginate solution (A2033, Sigma Aldrich, lot number: SLCF1478) prepared in Ca2 + −free DMEM (21,068,028, Thermo Fisher Scientific) to yield 1% alginate (16) with 55 μL/mL ECL, which is equal to a final cell density of 3 × 10^6^ cells/mL. Generated alginate microcapsules were collected in a 100 mM CaCl_2_ solution.

### 2.2. Serum-free culture for differentiation of C2C12 cells as monolayer and in 3D alginate beads

3 different serum-free media, i) AIM-V (A3830801, Thermo Fisher Scientific, United States), ii) DMEM with 1% B27 (17,504,044, Thermo Fisher Scientific, United States), iii) AIM-V with 1% B27, and serum-supplemented media as control iv) DMEM with 2% Horse Serum (H1270, Merck, Germany) were prepared for the differentiation of C2C12 cells. 90% of confluent myocytes C2C12 cells were harvested after dissociation using TrypLE and resuspended in serum-free media. For monolayer 2D culture, the 96 (100 uL/well) or 6 wells plates (1 mL/well) (treated for increased cell attachment, VWR, United States) were coated with ECL solution (5 uL/mL in DMEM medium) for 2 h at room temperature and subsequently washed with PBS followed by air-drying. Cells were seeded at a density of 3×10^5^ cells/cm2 and media was replaced every second day. For the alginate microcapsule 3D culture, the alginate beads were transferred into either a 96-well plate or a 6-well plate (TC-treated VWR, United States) and CaCl2 was removed and replaced by 200 μL or 3 mL of culture media, respectively. Alginate-encapsulated cells were cultivated on an orbital shaker plate (100 rpm) for 7 days in a humidified atmosphere with 5% CO_2_. The experimental methods for the observation of the morphology, viability, creatine kinase (CK) activity, and immunofluorescence microscopy is provided in the Supplementary material section.

### 2.3. Metabolites extraction procedures of 2D and 3D alginate microcapsule culture systems

The preparation of 2D and 3D alginate microcapsule cultures for the metabolomic analysis is described in the Supplementary material. Collection and extraction of the 2D and 3D alginate beads cell samples were performed on day 4 of cultivation and metabolite extraction of 2D cultured C2C12 cells was performed according to previous literature (13, 17). The extraction procedures of 3D alginate microcapsules were optimized (Supplementary Figure S3) and the final method is given as follows: Briefly, the alginate microcapsules were collected using a 100 μm sieve (CLS431752, cell strainer, Corning, United States) and then washed with 10 mL of cold 0.9% NaCl followed by cold MQ water. All microcapsules were collected in 10 mL of cold ACN:MQ (1:1, v/v) and immediately quenched in LN_2_. For the extraction of metabolites from the alginate microcapsules, 10–15 metal beads were added leading to the mechanical breakdown of the microcapsules. In total, three freeze–thaw cycles were repeated based on quenching in LN_2_ and thawing for the vortexing. After centrifugation (5 min, 5,000 rpm, 4°C), 9 mL of supernatant was collected and immediately quenched in LN_2_. After freeze-drying, dried cell extracts were reconstituted in 500 μL of MQ water and filtered through a spin-filter with a 3 kD cutoff by centrifugation (20 min, 13,000 rpm, 4°C).

### 2.4. Targeted MS-based metabolomics

Intracellular metabolites were quantified according to previous literature (17). TCA intermediates and phosphorylated metabolites were analyzed using capillary ion chromatography (capIC) coupled with triple quadrupole mass spectrometry (MS/MS) (18). O-benzyl hydroxyl amine and phenyl isothiocyanate were used to derivatize organic acids and amino acids, respectively, according to a previous publication (17). The processing of the data is described in Supplementary material section 1.5.

### 2.5. Statistical analysis

All results are provided as the average ± standard deviation (SD). Multivariate statistical analysis was performed in Metaboanalyst (19) after normalization of log transformation and auto-scaling. Independent student’s *t*-test was applied to compare the average between the two groups. The one-way ANOVA with posthoc Tukey HSD test was applied for the comparison of multiple groups. If the data were not normally distributed, non-parametric tests such as Mann–Whitney (two groups) or Kruskal-Wallis test (multiple groups) were performed using the SPSS version 27 program.

## 3. Results

### 3.1. Optimization and characterization for the establishment of alginate-C2C12 cells microcapsule culture

The voltage of the electrostatic droplet generator was optimized and two commercial cell attachment factors (gelatin and ECL) were tested to establish robust production of alginate microcapsules. A voltage of 5–5.25 kV for droplet generation was applied, resulting in spherical microcapsules and a homogenous size distribution (±2–3%) (Supplementary Table S1). Furthermore, we explored the incorporation of gelatin and ECL into the alginate as a cell adhesion matrix in both serum (DMEM+FBS) and serum-free media (AIM-V), since alginate lacks peptide sequence that supports cell attachment. Overall, encapsulated C2C12 cells in alginate microcapsule cultured in serum-free media showed a decreasing trend in viability, but no significant difference, compared to that cultured in serum-supplemented media, demonstrating the potential of serum-free media for the alginate microcapsule cultivation. A significant difference in cell viability between the addition of two extracellular matrices (ECL vs. Gelatin) into alginate was observed. Alginate beads with ECL showed significantly higher cell viability than alginate alone and alginate with gelatin in both serum-supplemented and serum-free media cultivation (Supplementary Figure S1). Finally, the addition of ECL was chosen to enhance cell survival in alginate microcapsule culture systems.

**Figure 2:**
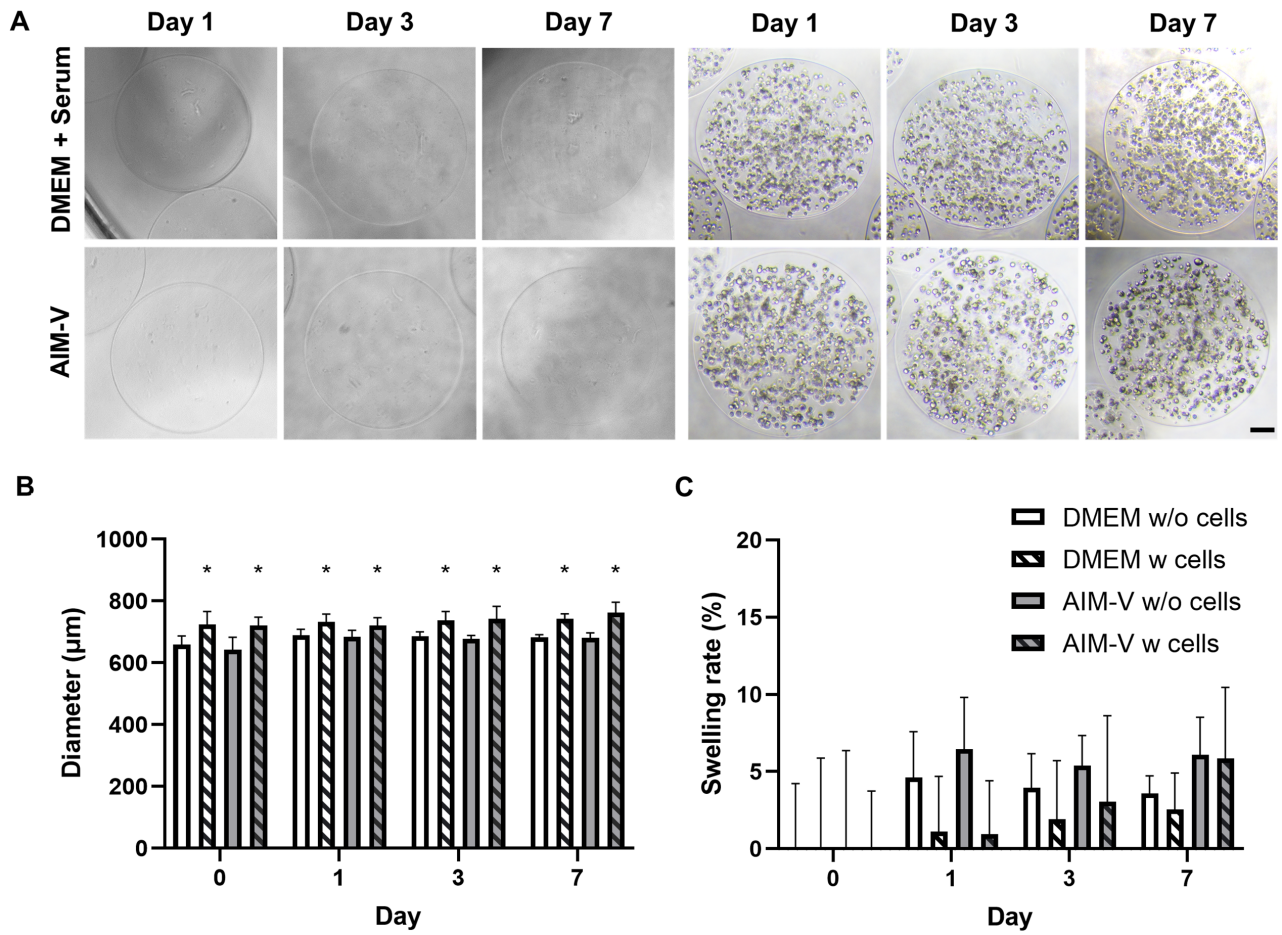
Characterization of final established alginate microcapsules with (W) and without (w/o) C2C12 cells in both Serum-supplemented (DMEM) and Serum-free (AIM-V) media 7 days of cultivation. **(A)** Representative phase contrast images, **(B)** the diameter of alginate microcapsules, * indicates significant differences alginate beads between with (w) and without (w/o) encapsulated C2C12 and **(C)** Swelling rate (%) during the cultivation period. Swelling rates of alginate microcapsules were normalized to the initial encapsulation culture day (Day 0) (*n*) = 10. Horse serum-supplemented (DMEM) and Serum-free media (AIM-V).

The final microcapsules produced after parameter optimization are presented in Figure 2A and were further characterized in terms of diameter size and swelling rate (%) of alginate microcapsules over 7 days of cultivation (Figures 2B,C). The exposure of encapsulated cells to extensive swelling induced by ion exchange and water uptake can reduce cell viability due to mechanical stress in the form of shear stress (20). In our case, extensive swelling was observed during the first 8 min after media exchange (data not shown). Therefore, the characterization of all microcapsules was performed after swelling equilibration (Figure 2A). Morphologically, all alginate microcapsules maintained a symmetrical and spherical shape for 7 days (Figure 2A). Only alginate microcapsules containing C2C12 cells showed a significantly enlarged diameter size (740 ± 40 μm) compared to microcapsules without cells (680 ± 15 μm). There was no significant difference in diameter observed between the two media types media (DMEM and serum-free AIM-V). Furthermore, the swelling rate of our beads was less than 10%, which suggests no degradation during 7 days of culture. Overall, the final alginate beads generated with the ECL attachment factor displayed a homogenous size distribution, spherical shape and low swelling rate, indicating that our established alginate microcapsule generation method is robust and reproducible for the cultivation of C2C12 cells under serum-free conditions.

### 3.2. Viability and differentiation assessment of C2C12 cells in 2D and 3D cell culture systems

**Figure 3:**
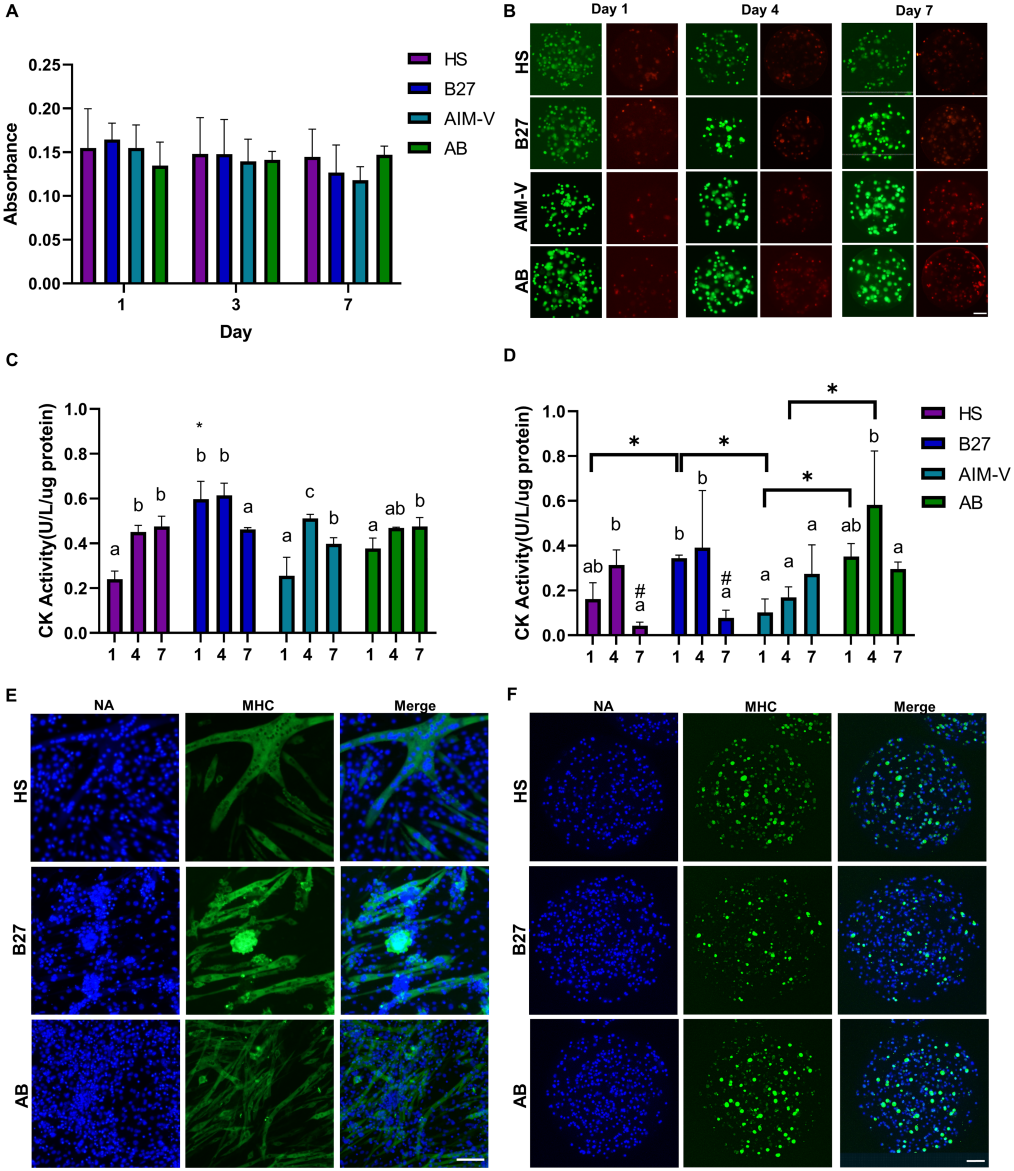
Viability and Differentiation assessment of Alginate-C2C12 cells microcapsules. **(A)** Cell viability of C2C12 cells in alginate microcapsules based on MTT assay was assessed for 7 days in 4 different media (*n*) = 3. **(B)** Representative images of Live/Dead (Green/Red) cell staining in alginate microcapsules. Live/Dead staining was performed on the 1st, 4th, and 7th day of cultivation. Creatine kinase (CK) activity was measured to evaluate myogenic differentiation during 7 days of cultivation for **(C)** 2D cultures and **(D)** alginate microcapsule cultures. The different letters indicate a significant difference in the same culture group between culture periods. *in **(C)** indicates a significant difference on day 1 versus all culture groups. *in **(D)** reveals a significant difference between indicating groups on the same day (*p* < 0.05). Representative images of immunocytochemical staining of MHC II (green) in **(E)** 2D cultures and **(F)** alginate microcapsule. Projection images after overlapping all Z-axis images were finally presented. Nucleic acids (NA) staining is indicated by the blue color. All scale bars indicate 100 μm (HS; DMEM+2% horse serum, B27; DMEM+1% B27, AIM-V, AB; AIM-V + 1% B27).

After establishing the alginate microcapsule culture system, monolayer (2D) and alginate microcapsules (3D) were further cultivated in the 4 different media conditions and evaluated in terms of viability and differentiation (Figure 3). 2D-cultured C2C12 cells differentiated into myotubes on the 4th day of cultivation and continued to generate multiple myotubes until 7 days. However, some detached cells were observed in the case of the DMEM-based media cultures (HS, B27; Supplementary Figure S2A). AB culture presented aligned myotubes accompanied by twitching movements, whereas DMEM-based cultures showed randomly distributed myotubes. Cell viability was evaluated by the MTT assay, and both 2D and 3D alginate microcapsules cultures showed stable cell viability indicated by steady absorbance throughout the 7 days of cultivation in HS, B27, and AB cultures (Figure 3A; Supplementary Figure S2). Additionally, Live/Dead assay was conducted to monitor the distribution of live and dead cells inside the alginate microcapsules (Figure 3C). Notably, a small number of dead cells were mainly found at the periphery of the capsules and not in the core.

After confirming cell survival for 7 days, we first assessed the differentiation by measuring CK activity in both 2D (Figure 3C) and 3D alginate microcapsule cultures (Figure 3D). The highest CK activity was observed after 4 days of cultivation with a similar range (0.4–0.6), and it significantly decreased on the 7th day in both culture modes. Surprisingly, 3D culture in AIM-V media showed the lowest CK activity throughout the culture period, suggesting that differentiation was not occurring. Therefore, we further evaluated myogenic differentiation by the immunostaining of MHC on 4th day of cultivation, except for AIM-V culture. In the case of 2D culture (Figure 3E), linear fiber types of multiple myotubes were predominantly formed in all culture media, while cluster types of differentiated myotubes were discovered in B27. Interestingly, varying diameter sizes of myotubes were shown, with the thickest diameters observed in HS (40 ± 19 μm), followed by B27 (22 ± 8 μm), and AB (11 ± 5 μm). MHC II expression levels were only significantly different between AB and HS cultures (1.2 fold increase; Supplementary Figure S2C). Regarding 3D alginate microcapsule cultures (Figure 3F), differentiated cells formed spheroids rather than linear-shaped fibers, and none of C2C12 cell spheroids exhibited twitching movements. Nucleic acid staining revealed that the cells are equally distributed within the alginate-ECL beads, and immunostaining identified cells positive for MHC in all cultures, confirming the myogenic differentiation. However, AB culture presented the highest MHC expression rate compared to both HS (1.5 fold increase) and B27 groups (2.1 fold increase; Supplementary Figure S2D).

### 3.3. Metabolite profiling between 2D and 3D alginate microcapsule culture

Before performing intracellular metabolic profile analysis, the extraction procedures for C2C12 cells cultured in alginate beads were optimized (Supplementary Figure S3). When using a ACN/MQ solvent with metal beads together with three freeze-thawing cycles under continuous vortexing, alginate beads were readily broken down and the energy charge was the highest value (0.87) among the 4 different conditions. Energy charge should be maintained between 0.7 and 0.95 within physiological range and stabilized at a value close to 0.9 (21). Our experimental results showed that the energy charge in all groups was within a physiological range and close to 0.9 (0.83–0.87) (Supplementary Table S3), indicating the energy state was stable during the microcapsule culture and extraction procedures.

**Figure 4:**
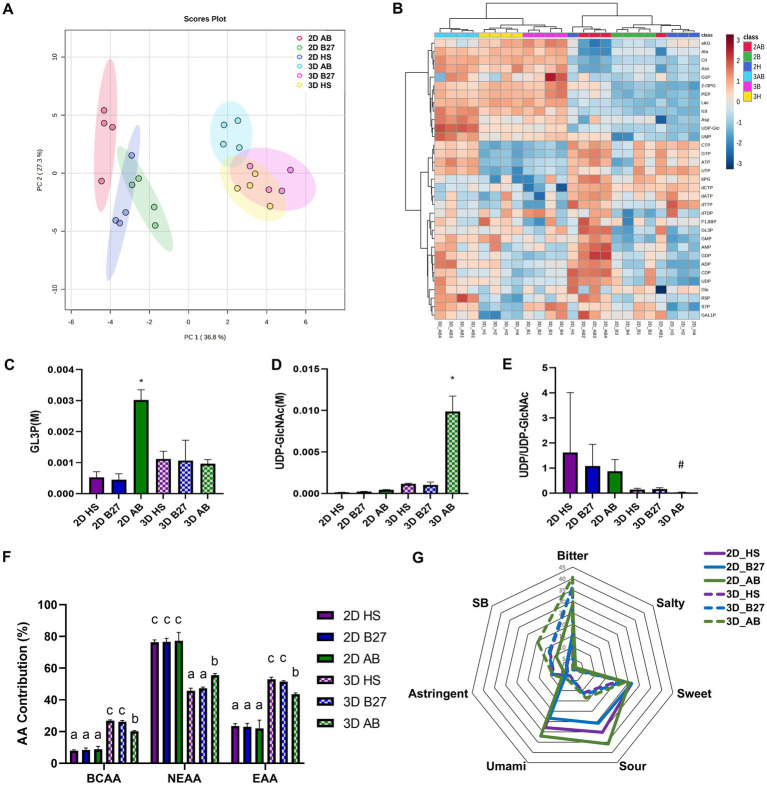
Metabolomic analysis of C2C12 cells and alginate microcapsules cultured in Serum-supplemented and Serum-free media (2D and 3D indicate monolayer culture in the petri dish and alginate microcapsule culture, respectively). Multivariate statistical analysis of intracellular metabolites data-set. **(A)** Score plot of Principal component analysis (PCA) with the first (PC1 presenting 36.8% of total variance) against the second (PC2 presenting 20.36% of total variance) principal component, which covers 64% of the total variance. The colored circle area reveals the 95% confidence interval of each group. **(B)** Heat map visualization and hierarchical cluster analysis (HCA) for intracellular metabolites were performed based on euclidean distance and wald clustering method. The red and blue squares in the heat map present high and low metabolite expression, respectively. Red; 2D culture in AB, Green; 2D culture in B27, Blue; 2D culture in HS, Light Blue; 3D culture in AB, Pink; 3D culture in B27, Yellow; 3D culture in HS. The absolute intracellular concentration of **(C)** GL3P, **(D)** UDP-GlcNAc, and **(E)** the ratio of UDP/UDP-GlcNAc, indicating O-GlcNAcylation. # indicates significant difference versus 2D HS, B27, AB cultures (*p* < 0.05). Analysis of contribution (%) of each amino acid to the total amino acid pools. **(F)** The contribution of BCAA (BCAA; branched-chain amino acids, EAA essential amino acids, NEAA; non-essential amino acids). **(G)** The distribution of tastes of monolayer cultured C2C12 cells and alginate-C2C12 cells microcapsule based on the amino acids contribution. The six different tastes were calculated based on previous publications (22) (Bitter; Proline + Phenylalanine + Valine + Leucine + Tryptophan + Arginine + Lysine, Salty; Aspartate, Sweet; Alanine + Proline + Valine + Lysine + Glycine, Sour; Aspartate + Glutamate + Lysine, Unami; Aspartate + Glutamate, Astringent; Lysine, Suppressing bitterness (SB); Arginine + Lysine).

After optimization of the extraction protocol, 53 metabolites were quantified in total (Supplementary Table S4). Unexpectedly, an enriched matrix was found in the alginate beads (Supplementary Table S5), which was identified as mostly organic acids (Pyruvate, Lactate, succinate, malate) and amino acids. Therefore, intracellular metabolite concentration was calculated after the subtraction of alginate matrix components. Finally, 33 intracellular metabolites could be quantified and multivariate statistical analyses were performed. The unsupervised statistical analysis of both principle component analysis (PCA; Figure 4A) and hierarchical cluster analysis (HCA; Figure 4B) revealed greater metabolic differences based on culture modes (2D vs. 3D alginate microcapsule) as opposed to culture media (Serum vs. Serum-free media). Interestingly, B27 and HS groups were more metabolically similar than AB and B27 groups, despite the fact that they share B27 supplementation. Among 33 intracellular metabolites, 13 metabolites showed a significant difference between the culture modes (2D vs. 3D alginate microcapsule; Supplementary Table S6). Specifically, concentrations of intermediates of glycolysis and TCA cycle, as well as lactate, were found to be higher in the 3D alginate microcapsule compared to 2D cultures, while nucleotide phosphates-related metabolite concentrations were lower.

Regarding the evaluation of myogenic differentiation using metabolites sets, three metabolites (GL3P, UDP-GlcNAc, and ratio of UDP to UDP-GlcNAc) in 2D and 3D alginate microcapsule cultures were compared (Figures 4C–E) as they have been previously proposed as markers of myotube differentiation (13). Surprisingly, only AB cultures showed significantly higher levels of GL3P in 2D and UDP-GlcNAc and the lowest rate of O-GlcNAcylation (only significant difference between 2D cultures) in 3D alginate microcapsule cultures, confirming muscle differentiation in alginate microcapsule cultures based on the intracellular metabolite dataset.

Finally, considering that the entire alginate muscle cell microcapsule is an animal protein source belonging to the food category, the nutrient composition distribution was further investigated (Supplementary Figure S4). Among 8 metabolites classes, amino acids (AA) showed the most significant difference between culture modes (2D vs. 3D) and serum-free media (HS, B27 vs. AB in 2D and HS vs. AB in 3D), followed by lactate (2D vs. 3D, HS, B27 vs. AB in 2D), and nucleoside phosphates (only 2D vs. 3D). Therefore, we further investigated the nutrient composition, focusing on amino acids profiles, since amino acids not only comprise proteins but also significantly contribute to the taste and flavor of the meat (22). Alginate-C2C12 cells microcapsules contained a significantly higher contribution of branched-chain amino acids (BCAA) and essential amino acids (EAA) than 2D cultured C2C12 cells (Figure 4F), which might be derived from the alginate microcapsule matrix. In addition, only alginate microcapsule cultures showed a significant difference in AA composition between culture media (HS, B27 vs. AB), but no change was observed in 2D cultures. Furthermore, we attempted to determine the 6 different tastes based on previous publications using amino acids profiles (22) (Figure 4G). Bitterness is mainly prevailing in alginate microcapsules, while umami and sour flavors are more prevalent in monolayer cultured cell products.

## 4. Discussion

In this study, we established serum-free microcarrier culture system using edible alginate in encapsulation for the differentiation of mouse myoblast cells (C2C12 cells) and demonstrated its potential as a model of an alternative animal protein source. The system was evaluated based on viability, differentiation, and metabolic profiling. Although alginate is edible and ideal for cell encapsulation, it lacks cell adhesion motifs and requires the inclusion of specific attachment domains, such as RGD or YIGSR sequence. However, the variability of laboratory strategies required to achieve this may limit accessibility for researchers lacking necessary resources and expertise (23, 24). Therefore, we focused on establishing alginate microcapsule culture using only readily accessible commercial sources, including cell attachment factors and media.

Optimizing alginate microcapsule generation procedures revealed that combining alginate with ECL matrix resulted in higher cell viability compared to using gelatin or alginate alone. This finding is surprising, given that previous studies reported successful cultures of C2C12 myotubes using gelatin in the form of micromold (25) and fibers (26), as well as alginate-gelatin microcapsule culture for the maturation of human myelomonocytic cell line (16). Gelatin is typically obtained from high temperature-treated or hydrolyzed collagen type I and possesses the RGD sequence (27), whereas ECL is a mixture of three extracellular matrix proteins: entactin (28), collagen IV, and laminin (23), and all of which contain cell adherent peptide sequences. The greater viability observed in alginate-ECL microcapsule could be explained by a previous study showing that the laminin significantly supported myotube formation (2-fold increase) from C2C12 and human skeletal muscle compared to fibronectin, collagen I, and collagen type IV (29).

The final alginate-ECL beads had average diameters of 740 μm, which was within our target size range based on a previous publication recommending bead diameters between 600 μm and 1,000 μm for optimal bead quality and reduced handling errors (30). The final swelling rate during 7 days of cultivation was determined to be less than 10%, which is consistent with the range reported in a previous study (16). C2C12 cells cultured in alginate-ECL microcapsules remained viable for 7 days without the necrosis in the core of the microcapsule, which can occur due to insufficient exchange of nutrients and oxygen (31). These results indicate that the microenvironment of our generated microcapsules is sufficient for cell survival and maintenance.

While there were no dramatic differences in morphological characteristics and viability between serum-supplement and serum-free cultures, myogenic differentiation was more influenced by the different media. Unexpectedly, the CK activity of C2C12 cells cultured in AIM-V media did not increase during the cultivation and showed the lowest CK activity in 3D microcapsule cultures. AIM-V media was originally developed for T-cell proliferation cultivation and may require a serum substitute for myogenic differentiation, especially in the 3D culture. Moreover, in the case of 2D monolayer culture, the thickest myotube was observed in HS followed by B27 and AB. These results are consistent with previous findings in our serum-free 2D culture system (13), highlighting the reproducibility of our experimental approach. However, further investigation is needed to determine how morphological differences in differentiation between culture modes (2D fiber versus 3D spheroid) impact the metabolism.

To the best of our knowledge, this is the first study to investigate the metabolic profiles of skeletal muscle cells in 2D and 3D alginate microcapsule cultures, including the optimization of extraction procedures. Although a few procedures for extracting metabolites from 3D mammalian cell cultures have recently been reported, they have been studied only in matrix-free 3D spheroid platforms (14, 32, 33). Fortunately, we were able to freeze the alginate microcapsules in liquid nitrogen and then break the capsules with physical treatments (metal beads and vortexing) to effectively extract the metabolites. Although we also attempted to use glass beads, it was impossible to crack alginate beads (data not shown). Moreover, the suitability of our extraction method was evidenced by energy charge, which was shown to be (0.83–0.87) in all culture experimental groups, indicating that not only our established alginate microcapsules culture but also the extraction procedure is reproducible and the survival of cells was granted.

Although we attempted to analyze a comprehensive range of intracellular metabolites, our analysis was limited to only 33 metabolites due to the unexpected detection of alginate microcapsule-derived compounds. The majority of these metabolites were identified as organic acids and amino acids, likely originating from alginate (34) and the culture media used to dissolve it. Nevertheless, our results suggest that differences in intracellular metabolism are more dependent on the culture mode than on culture media. Interestingly, we observed a greater similarity in the metabolic phenotypes between cells cultured in HS and B27 compared to those cultured in B27 and AB, implying that the metabolic phenotype is more strongly influenced by basic media (DMEM vs. AIM-V) rather than the supplementation used.

Higher levels of glycolysis and TCA intermediates, as well as lactate, were observed in 3D alginate microcapsule compared to 2D culture. These results are in line with a recent study reporting similar findings of elevated metabolites involved in glycolysis and TCA cycles in 3D spheroid compared to 2D culture, suggesting that energy is produced through both mitochondria simultaneously with aerobic glycolysis in 3D spheroid culture (35). Moreover, a previous study reported that increased expression of MCT1 (Monocarboxylate transporter), a lactate transporter, leads to greater lactate uptake in rat and human skeletal muscles (36). Intriguingly, another study demonstrated that protein expression of MCT1 was significantly elevated in 3D spheroid cultures (32), which might explain the observed increase in intracellular levels in 3D alginate in our study.

In addition, our analysis revealed that amino acid composition was the most distinguishable nutrient, suggesting that specific media or culture modes could be selected to achieve the desired amino acid composition. Furthermore, since essential amino acids (EAAs), including branched-chain amino acids (BCAAs), must be obtained from the diet, ingesting them in the form of alginate-C2C12 cell microcapsules, rather than just cell products, could have potential health benefits. Lastly, we attempted to evaluate myogenic differentiation and predict taste using metabolite sets, indicating that metabolite profiles could be suggested for quality control assessment in the production process of artificial protein. However, further studies are necessary to determine the association between metabolite profiles and taste evaluation.

In this study, we successfully established alginate microcapsules in flask lab-scale culture for metabolomics analysis, providing evidence of the feasibility of scaling up to bioreactor culture in the future. Additionally, since our study only focused on the differentiation of skeletal muscle in the absence of serum, further studies including the proliferation process should be considered.

## 5. Conclusion

Alginate-muscle cells microcapsules in serum-free culture platform have been successfully established for the differentiation of murine skeletal muscle C2C12 cells using only cost-effective commercially available products with the advantage of being readily accessible to the researchers. We demonstrated the robustness and reproducibility of our culture system, evidenced by low variation in size, swelling rate (<10%), and energy charge. Cells survived for 7 days in the alginate beads without core necrosis. AB culture showed the highest myogenic differentiation rate and the most distinct metabolite profiles. Furthermore, metabolic phenotypes are more dependent on culture modes than culture media, and basic media than the serum substitute. We believe that this research contributes to the development of alternative animal protein source in the food industry and pave the way for the scalability of the production process.

## Data availability statement

The original contributions presented in the study are included in the article/Supplementary material, further inquiries can be directed to the corresponding authors.

## Author contributions

JS: writing the initial manuscript and performing and analyzing the experiments. PB: scientific discussion and financial support. JK: technical and scientific support for alginate-encapsulation procedures. MJ: conceptualization, initial writing, and editing the manuscript, performing and analyzing the experiments, and metabolomics data. All authors contributed to the article and approved the submitted version.

## Funding

This work was funded by the NTNU Strategic Biotechnology Program.

## Conflict of interest

The authors declare that the research was conducted in the absence of any commercial or financial relationships that could be construed as a potential conflict of interest.

## Publisher’s note

All claims expressed in this article are solely those of the authors and do not necessarily represent those of their affiliated organizations, or those of the publisher, the editors and the reviewers. Any product that may be evaluated in this article, or claim that may be made by its manufacturer, is not guaranteed or endorsed by the publisher.
