# Supplementary material for: Serum-free alginate-C2C12 cells microcapsule as a model of alternative animal protein source
Source: Front Nutr. 2023 May 11;10:1184178. doi: 10.3389/fnut.2023.1184178 (PMC10213942; doi:10.3389/fnut.2023.1184178)
Supplement: Supplementary file 1 [file Data_Sheet_1.docx]

Supplementary Material

Serum-free alginate-C2C12 cells microcapsule as a model of alternative animal protein source

**Jana Scheffold^1^, Per Bruheim^1^, Joachim Sebastian Kjesbu^1^, Mi Jang^1^***

*** Correspondence:**

**Mi Jang**

[kia3111@gmail.com](mailto:kia3111@gmail.com)

# Supplementary materials and methods

**1.1 Cultivation and maintenance of C2C12 cells**

C2C12 cells (ATCC CRL-1771, lot number: 70024392) were cultivated in DMEM medium (D6429, Merck, Germany) supplemented with 10% FBS (F7524, Merck, Germany, lot number: BCBX9154) and 1% penicillin-streptomycin (P/S) (15140122, Thermo Fisher Scientific, USA) at 37°C under a humidified atmosphere with 5% CO2 (HERAcell 150i, Thermo Fisher Scientific, USA) in T-75 or T-175 flasks (Surface Treated, VWR, USA). For the subculture, the cells were detached upon reaching 60% confluency using TrypLE (12604013, Gibco BRL Life Technologies), which is a substitution of trypsin for the serum-free culture, as its inactivation does not require FBS incubation. The medium was renewed every 2-3 days. Cells at passage numbers 3-6 were used for the experiments

**1.2 Morphology and Cell viability**

The cell morphology was monitored using a bright field phase-contrast microscope (Eclipse Ts2, Nikon, Japan) and the NIS Elements F 4.51 software. Cell viability was assessed using the MTT assay based on a previous publication (Saberianpour et al. 2019). 20 µl of MTT solution (final concentration of 0.5 mg/mL) was added to each well in a 96-wells plate and incubated for 2 h and 4 h at 37°C for 2D and 3D alginate microcapsule cultures, respectively. The DMSO solution was replaced after removing the solution and incubated for 30 min to dissolve the purple formazan product. The optical density was measured using a microplate reader (Spark 20 M, Tecan, Switzerland) at 570 nm. Additionally, Live/Dead staining (R37601, Thermo Fisher Scientific) was performed to assess the viability of encapsulated cells according to the manufacturer's instructions. At least three beads from each condition were used. Fluorescent images were taken with a fluorescent microscope (Eclipse TS2, Nikon) and the NIS Element F 4.51 software (Nikon).

**1.3 CK activity measurement.**

For cell recovery in alginate microcapsules, alginate beads were first collected from 6-well plate using a 100 μm sieve (CLS431752, cell strainer, Corning, USA), washed three times with PBS, and then incubated in solubilization solution (5 mM EDTA (EDS-100G, Sigma Aldrich), 150 mM NaCl (27810.295, VWR) and 10 mM HEPES (H0887, Sigma Aldrich)) for less than 10 min. After brief centrifugation (1000 rpm, 3 min), cell pellets were resuspended in 200 μLof lysis buffer according to the previous literature (Jang et al. 2022). The lysed cell solution was centrifuged (4°C, 13000Xg, 10 min), and the extracted protein supernatant was stored at -20°C for the further measurement of protein content. Creatine kinase activity was measured according to the manufacturer´s instruction (MAK116, Sigma Aldrich) with slight modifications. Briefly, 5 μL and 25 μL of protein extract were added to a 96-well plate for 2D and 3D alginate microcapsule culture samples, respectively. Then 100 μL of reconstituted reagent was added to each sample and incubated at 37°C. The absorbance at 340 nm was measured every 5 minutes for 40 minutes using a microplate reader (Spark 20 M, Tecan, Switzerland). For 2D samples, the CK activity was calculated using the absorbance after 10 and 15 min, $CK activity=\frac{{{(A}_{340})}_{15 min}-{{(A}_{340})}_{10 min}}{{{(A}_{340})}_{cal}-{{(A}_{340})}_{blank}}*600$, and for 3D samples the absorbance after 20 min and 40 min, $CK activity=\frac{{{(A}_{340})}_{40 min}-{{(A}_{340})}_{20 min}}{{{(A}_{340})}_{cal}-{{(A}_{340})}_{blank}}*150$. The final CK activity was normalized by the total protein content, which was determined using the BCA protein assay kit (23225, Thermo Fisher Scientific). The final CK activity was normalized by the total protein content, which was determined using the BCA protein assay kit (23225, Thermo Fisher Scientific).

**1.4 Immunofluorescence microscopy**

In the case of 2D culture, cells were fixed in 4% paraformaldehyde (PFA) solution for 15 min and permeabilized in 0.1% Triton X-100 prepared in PBS buffer. After blocking with 1% BSA in TBST for 1 hour, an Alexa fluor conjugated MHC antibody (53-6503-82, myosin 4 monoclonal antibody MF20, Thermo Fisher Scientific, 1:100) was incubated for 1 h at room temperature, and then washed with PBS. For encapsulated cells in alginate for 3D culture, at least three beads from each condition were collected for staining. A syringe with a 100 μm needle (BD microlance 3 30Gx ½”, Becton Dickinson, USA) was used during all washing steps, and a washing solution (150 mM NaCl, 5 mM CaCl2, 20 mM HEPES, pH 7.4) was used for the 3D samples instead of PBS. The alginate microcapsules were fixed using 4% PFA, followed by permeabilization (0.1% Triton X-100 in washing solution, 15 minutes) and blocking (1% BSA in washing solution, 1 h). Finally, a conjugated MHC antibody was incubated for 1 h at room temperature. Nucleic acid was counterstained using 5 μL/mL Hoescht 33342 (H3570, Thermo Fisher Scientific) for both cultures. Fluorescent images were taken using a fluorescent microscope (Eclipse TS2, Nikon) and the NIS Element F 4.51 software (Nikon) and a fluorescent confocal laser scanning microscope (Leica DMi8 Confocal Microscope, Ger) coupled with the Leica LAS-X software, for monolayer 2D and 3D alginate microcapsule cultures, respectively. The MHC positive area was quantified and normalized to the nucleic acid-stained area using Image J software. 5 randomly selected images were quantified including Figures 3E and 3F. The final data are presented as a fold change compared with the average of the HS group in 2D and 3D alginate microcapsule culture, separately.

**1.5 Preparation for 2D and 3D alginate microcapsule culture systems for metabolite profiling**

To prepare for metabolite profiling of monolayer 2D and 3D alginate microcapsules culture samples, cells were cultivated under 3 different media conditions (DMEM with 2% HS, DMEM with 1% of B27, and AIM-V with 1% B27) and harvested on 4th days of cultivation. In the case of 2D culture, cells were seeded at 3x105 cells/cm2 into ECL-coated 90 mm culture plates (increased cell attachment, VWR) and 6 wells plates for metabolic profiling and cell counting, respectively. For alginate microcapsule culture, 3x106 cells were encapsulated in 1 mL of alginate solution for one replicate and cultivated in 3 ml of media in a T25 flask (surface-treated). In total, 4 flasks were prepared, and 200ul of collected beads were solubilized according to the method section above for the cell counting. The number and volumes of cells were measured using the MoxiZ cell counter.

**1.6 Data processing**

Masslynx 4.1 (Waters Corporation, USA) and its TargetLynx application was used for the processing of all MS-based data. Intracellular metabolite concentrations were corrected for dilution, and sample concentration and normalized by cell number and cell volume.

# Supplementary Figures and Tables

**Supplementary Table 1**. Respective voltage of the electrostatic droplet generator (NTNU in-house fabrication), morphology, and diameter size of beads were described. 10 beads from each condition were randomly monitored. Initial trials with sodium alginate (W201502-sample, Sigma-Aldrich, lot number: MKCH7131) were used to evaluate the compatibility and reproducibility of the alginate microcapsule generation with commercial ECM materials. A 3% pure alginate solution dissolved in Ca-free PBS, 3% alginate + 5 μL/mL ECL (ECL attachment matrix, 08-110, Sigma Aldrich), and 2.4% alginate + 0.1 % gelatin (80:20 (v/v)) were tested. After confirming that the alginate beads were generated in a symmetric circular shape with low size variation, 1% of medium viscosity alginate dissolved in Ca2+-free DMEM was used for the entire experiment.

| **Alginate**  **Concentration** | **Voltage [kV]** | **Bead morphology** | **Average diameter [μm]** |
| --- | --- | --- | --- |
| **Initial trial: Alginate dissolved in Ca^2+^-free PBS** | | | |
| **3% alginate** | 7.00 | round shape, no tailing | 386 ± 27 |
|  | 6.25 | round shape, size variation, no tailing | 467 ± 38 |
|  | 6.00 | round shape, size variation, no tailing | 419 ± 33 |
|  | 5.75 | round shape, size variation, no tailing | 474 ± 33 |
|  | 5.50 | round shape, size variation, no tailing | 451 ± 15 |
|  | 5.25 | round shape, no tailing | 522 ± 8 |
| **3% alginate + ECL** | 6.80 | ellipse shape | 446 ± 14 |
|  | 6.00 | perfect round shape | 559 ± 95 |
|  | 5.75 | round shape | 609 ± 43 |
|  | 5.50 | perfect round shape | 631 ± 36 |
|  | 5.25 | perfect round shape | 667 ± 5 |
| **2.4% Alginate + 0.1% gelatin** | 7.5 | round shape | 414 ± 44 |
|  | 5.50 | perfect round shape, no tailing | 500 ± 12 |
| **Alginate dissolved in Ca^2+^-free DMEM** | | | |
| **1% alginate** | 5.50 | round shape, no tailing, size variation | 265 ± 10 |
|  | 5.00 | perfect round shape | 535 ± 10 |
|  | 4.50 | perfect round shape | 925 ± 18 |
| **1% alginate + ECL** | 5.50 | round shape, size variation | 318 ± 42 |
|  | 5.25 | round shape, size variation | 421 ± 35 |
|  | 5.10 | round shape | 485 ± 11 |

Supplementary Table 2. Chemical composition of medium viscosity alginate used in this study. Information was obtained by 1H-NMR and SEC-MALLS. Detailed information includes the G and M-content, the frequency of the building blocks, and the average molecular weight of alginate

| **F_G_** | **F_M_** | **F_GG_** | **F_MM_** | **F_MG/GM_** | **F_GGM/MMG_** | **F_MGM_** | **F_GGG_** | **N_G>1_** | **MW [kDa]** |
| --- | --- | --- | --- | --- | --- | --- | --- | --- | --- |
| 0.38 | 0.62 | 0.20 | 0.44 | 0.18 | 0.07 | 0.14 | 0.13 | 3.62 | 320 |

**Supplementary Figure 1**. MTT assay of encapsulated C2C12 cells in alginate beads with two different types of ECM (ECL and gelatin) for the measurement of cell viability at 1 and 3^rd^ day of cultivation. Serum-supplemented media (DMEM + 10% FBS) and serum-free media (AIM-V) were tested to evaluate the suitability of the alginate bead cultures for 3 days.


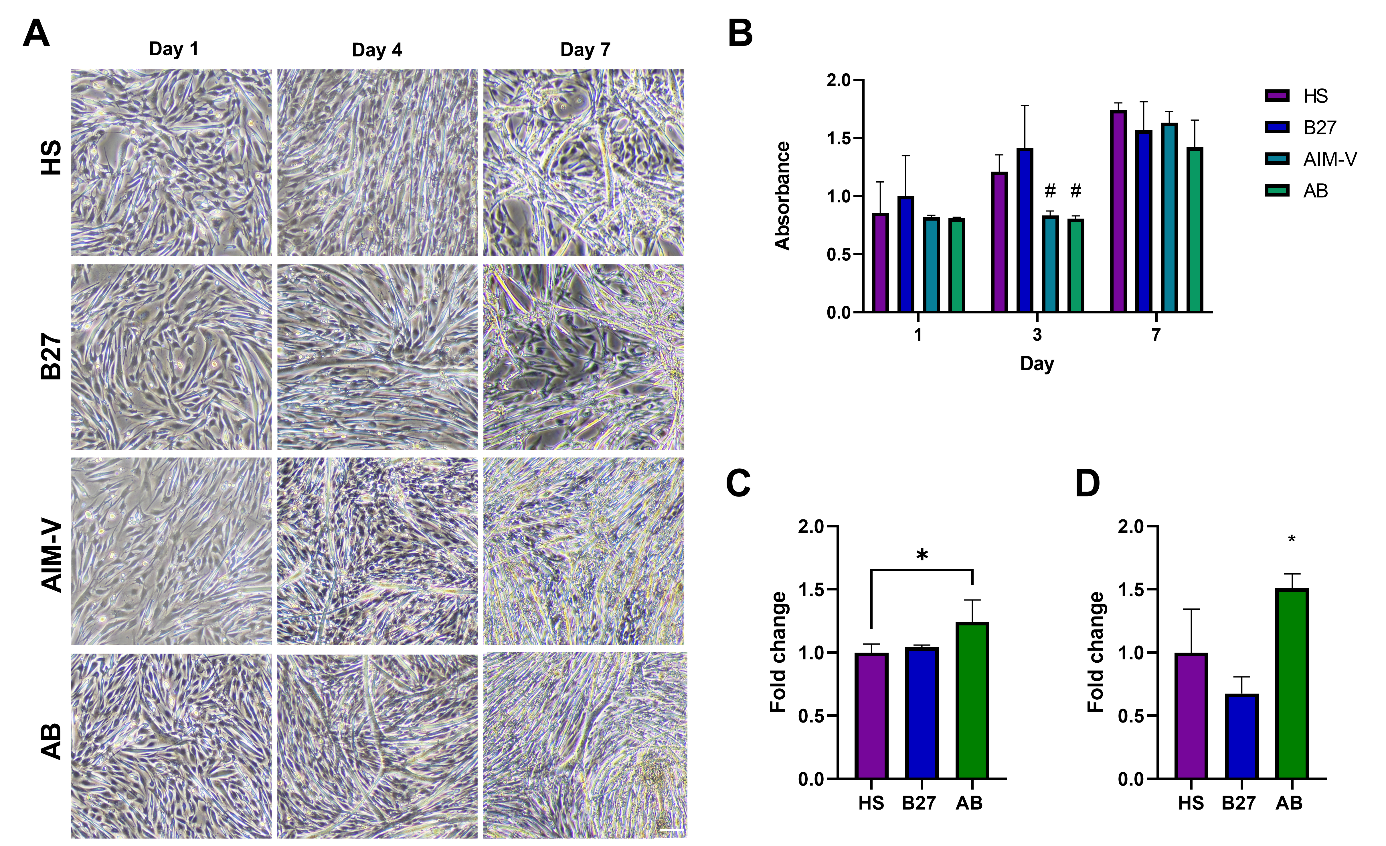


**Supplementary Figure 2.** (A) Representative images of cell morphology in 2D culture (monolayer) for 7 days. (B) Cell viability of 2D cultured C2C12 cells in 4 different culture conditions. MTT assay was performed for 7 days of cultivation. (n)=3. #p<0.05 versus B27 group. (C) Fold change of quantification of MHC in 2D culture. * indicates significant difference (<0.05) (n)=5. (D) Fold change of quantification of MHC in alginate microcapsules culture. * indicates significant difference between HS and B27 groups (p<0.05) (n)=5. (HS; DMEM+2% horse serum, B27; DMEM+1% B27, AIM-V, AB; AIM-V+1% B27)


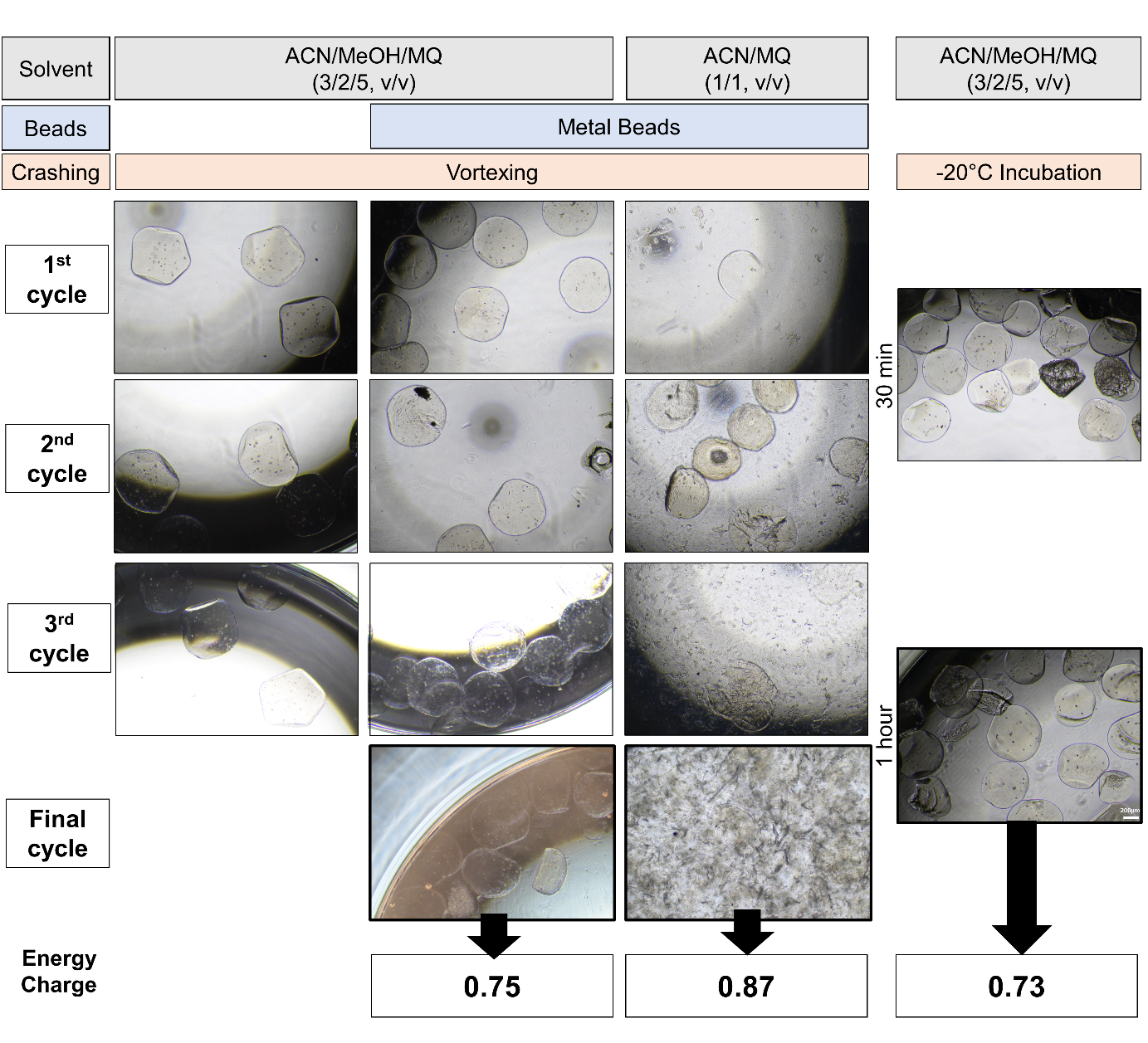


**Supplementary Figure 3**. Representative images of optimization of metabolites extraction procedures for the encapsulated alginate bead culture systems. 3 different parameters (different solvents, the existence of metal beads, crashing methods for alginate beads between continuous vortexing and low temperature (-20°C) incubation) were compared and the pictures were taken after each cycle of the freeze-thaw step to monitor the cracking status of alginate beads. Each cycle was performed with the addition of 10 metal beads and a continued vortexing procedure. The energy charge of selected three different extraction conditions (bold images) was calculated to evaluate the extraction quality for the intracellular metabolites profiling. ACN, MeOH, and MQ indicate Acetonitrile, Methanol, and Milli-Q water.

**Supplementary Table 3**. Energy Charge of 3 different alginate beads culture conditions performed based on established culture and extraction methods. (HS; DMEM+2% horse serum, B27; DMEM+1% B27, AIM-V, AB; AIM-V+1% B27). (n)=4.

| **3D alginate beads**  **culture condition** | **Energy Charge** |
| --- | --- |
| HS | 0.83±0.07 |
| B27 | 0.87±0.01 |
| AB | 0.86±0.05 |

**Supplementary Table 4**. List of analyzed metabolites for current study and abbreviations

| **Class** | **Abbreviation** | **Full name** |
| --- | --- | --- |
| Glycolysis | F1,6BP | Fructose 1,6-biphosphate |
|  | 2-/3PG | 2-/3-Phopho-D.glycerate |
|  | PEP | Phosphoenolpyruvic acid |
|  | Pyr | Pyruvic acid |
| Lac | Lac | Lactate |
| PPP  (Pentose phosphate pathway) | 6PG | 6-Phospho-D-Gluconate |
|  | R5P | D-Ribose-5-phosphate |
|  | S7P | D-Sedoheptulose-7-phosphate |
| Sugar phosphates | G1P | Glucose-1-phosphate |
|  | GAL1P | Galactose-1-phosphate |
|  | GL3P | Glycerol-3-phosphate |
|  | UDP-GlcNAc | Uridine diphosphate *N*-acetylglucosamine |
| TCA cycle | Cit | Citrate |
|  | ICit | Isocitrate |
|  | aKG | α-ketoglutarate |
|  | Suc | Succinate |
|  | Fum | Fumarate |
|  | Mal | Malate |
| NP  (Nucleoside phosphates) | AMP | Adenosine monophosphate |
|  | ADP | Adenosine diphosphate |
|  | ATP | Adenosine triphosphate |
|  | CDP | Cytidine diphosphate |
|  | CTP | Cytidine triphosphate |
|  | GMP | Guanosine monophosphate |
|  | GDP | Guanosine diphosphate |
|  | GTP | Guanosine triphosphate |
|  | UMP | Uridine monophosphate |
|  | UDP | Uridine diphosphate |
|  | UTP | Uridine triphosphate |
| Deoxy nucleoside phosphates | dATP | Deoxyadenosine triphosphate |
|  | dCTP | Deoxycytidine triphosphate |
|  | dTTP | Deoxythymidine triphosphate |
|  | dGTP | Deoxguanosine triphosphate |
| AA  (Amino acids) | Ala | Alanine |
|  | Arg | Arginine |
|  | Asn | Asparagine |
|  | Asp | Aspartate |
|  | Cys | Cysteine |
|  | Gln | Glutamine |
|  | Glu | Glutamate |
|  | Gly | Glycine |
|  | His | Histidine |
|  | Ile | Isoleucine |
|  | Leu | Leucine |
|  | Lys | Lysine |
|  | Met | Methionine |
|  | Phe | Phenylalanine |
|  | Pro | Proline |
|  | Ser | Serine |
|  | Thr | Threonine |
|  | Trp | Tryptophan |
|  | Tyr | Tyrosine |
|  | Val | Valine |

**Supplementary Table 5**. The concentration of alginate beads matrix. (n)=3

| **Class** | **Metabolites** | **Concentration (µM)** | **Class** | **Metabolites** | **Concentration (µM)** |
| --- | --- | --- | --- | --- | --- |
| **Glycolysis** | Pyr | 572±79 | **AA** | Gln | 1015±74 |
| **Lactate** | Lac | 559±60 |  | Glu | 55±5 |
| **PPP** | 6PG | 0.008±0.006 |  | Gly | 213±22 |
|  | R5P | 0.046±0.003 |  | His | 98±5 |
| **Sugar phosphate** | GL3P | 0.17±0.06 |  | Ile | 402±44 |
| **TCA cycle** | Cit | 4.7±2.2 |  | Leu | 408±28 |
|  | iCit | 1.1±0.5 |  | Lys | 433±54 |
|  | aKG | 0.03±0.02 |  | Met | 100±1 |
|  | Suc | 22±2.4 |  | Phe | 208±15 |
|  | Fum | 1.0±0.08 |  | Pro | 59±7 |
|  | Mal | 4.7±0.2 |  | Ser | 211±23 |
| **AA** | Ala | 66±7 |  | Thr | 346±49 |
|  | Arg | 327±37 |  | Trp | 35±4 |
|  | Asp | 0.2±0.03 |  | Tyr | 257±19 |
|  | Cys | 68±5 |  | Val | 456±59 |

**Supplementary Table 6.** Fold changes of intracellular metabolites in 3D alginate microcapsule to 2D cultures. Only significantly different metabolites were presented (p<0.05). Bold indicates a statistically significant difference below p<0.0005. Red and green colors indicate the increased and decreased levels of intracellular metabolites from C2C12 cells in alginate microcapsules compared to 2D culture, respectively. (n)=4.

| **Class** | **Metabolites** | **Fold change**  **(3D/2D)** |
| --- | --- | --- |
| Glycolysis | 2-/3PG | 4.2±1.8 |
|  | PEP | 10.4±5.7 |
| Lac | **Lac** | **18.9±5.0** |
| TCA cycle | **Cit** | **25.2±8.9** |
|  | Icit | 26.2±17.0 |
|  | aKG | 6.0±4.1 |
| NP | CTP | 0.5±0.2 |
|  | dCTP | 0.2±0.1 |
|  | GTP | 0.5±0.3 |
|  | UMP | 1.8±0.5 |
| AA | **Ala** | **6.8±1.9** |
|  | Asn | 8.0±3.7 |
|  | Asp | 2.8±1.4 |


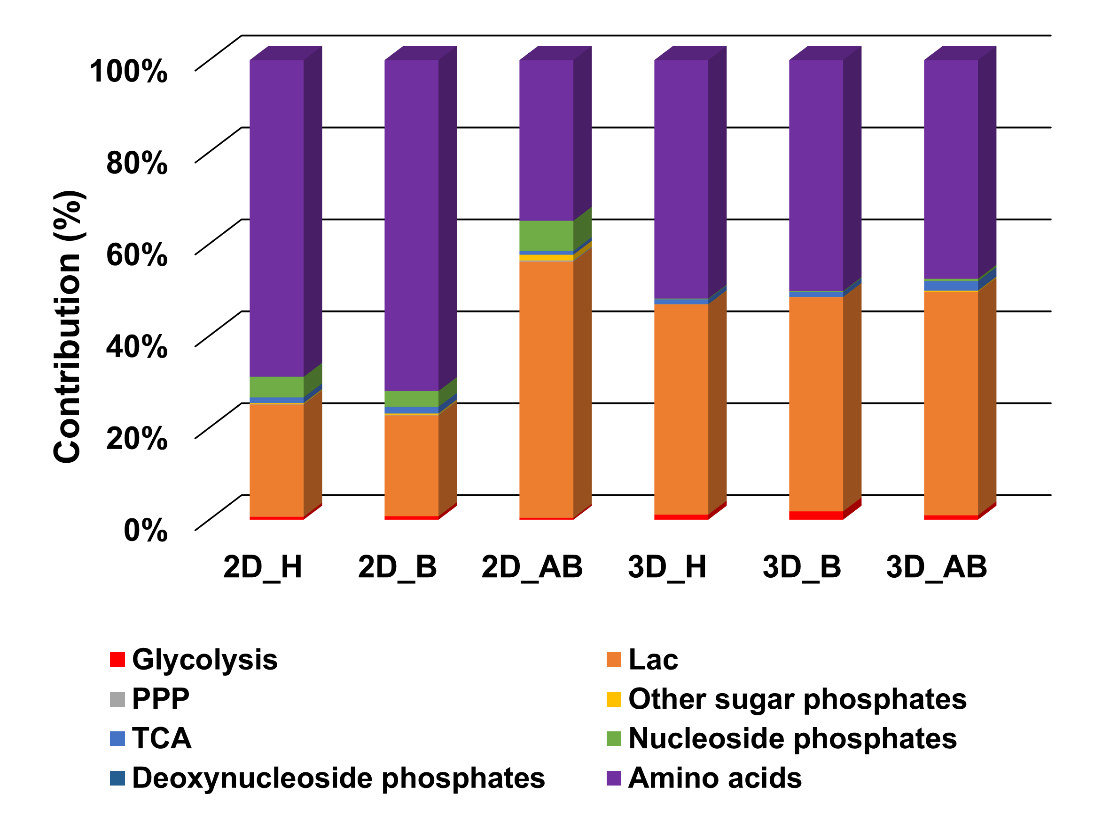


**Supplementary Figure 4**. Contribution (%) of 8 different metabolite classes to the total metabolite pools. (n)=4.
